# Three new species of *Dicephalospora* from China as revealed by morphological and molecular evidences

**DOI:** 10.3897/mycokeys.55.33859

**Published:** 2019-07-01

**Authors:** Huan-Di Zheng, Wen-Ying Zhuang

**Affiliations:** 1 State Key Laboratory of Mycology, Institute of Microbiology, Chinese Academy of Sciences, Beijing 100101, China Institute of Microbiology, Chinese Academy of Sciences Beijing China

**Keywords:** Morphology, phylogeny, species diversity, taxonomy

## Abstract

Three new species of *Dicephalospora* are introduced based on morphological characters and DNA sequence analyses (maximum parsimony and neighbor-joining methods), viz. *D.albolutea*, *D.shennongjiana*, and *D.yunnanica*. All of them lack mucilaginous caps at ascospore poles. *Dicephalosporaalbolutea* is distinguished by cream to yellowish white apothecia and slightly curved ascospores. *Dicephalosporashennongjiana* is characterized by yellow apothecia, elliptical-fusoid ascospores 19−22 × 7−8.8 μm, and J+ asci 130−150 × 14−16.5 μm. *Dicephalosporayunnanica* is distinguished by orange apothecia and fusoid ascospores 16.5−25.3 × 3.3−3.5 μm. Descriptions and illustrations of the new species as well as a key to the known species in the genus are provided.

## Introduction

*Dicephalospora* Spooner is a small genus established by Spooner (1987) with *D.calochroa* (Syd. & P. Syd.) Spooner as the type species. The poles of ascospores with a mucilaginous cap and J+ asci were treated as two important features to delimitate the genus, but a later study proved they are not reliable features at the generic level (Zhuang et al. 2016). The emended diagnostic characters of the genus are that apothecia erumpent or superficial, stipitate, yellow, orange, red to blackish, ectal excipulum of textura prismatica with refractive walls, medullary excipulum of textura intricata, asci J+ or J- in Melzer’s reagent, ascospores hyaline, subellipsoid to fusoid, guttulate, poles either with a mucilaginous cap or not, paraphyses filiform, straight or slightly curved at apex, and occurring on rotten wood, twigs, and leaf petioles (Zhuang et al. 2016). The genus was once treated as a member of Rutstroemiaceae (Kirk et al. 2008), Helotiaceae (Wijayawardene et al. 2017, 2018), or Sclerotiniaceae (Index Fungorum 2019). Including *Dicephalospora* in Helotiaceae is more reasonable in view of the phylogenetic studies of related groups in recent years (Han et al. 2014; Zhao et al. 2016).

Zhuang et al. (2016) carried out a comprehensive study on taxonomy of *Dicephalospora* in China and provided a key to the known species of the genus. Approximately, 10 species are currently accepted in the genus and nine of them have been found in China (Zhuang 1995a, 1995b, 1999; Verkley 2004; Zhuang et al. 2016). Dicephalosterol was discovered from the culture of *D.rufocornea* (Hosoya et al. 1999). This compound is a new testosterone 5α-reductase inhibitor and has a potential to be developed as a drug to prevent and cure prostatic hypertrophy (Hosoya et al. 1999). Additional information about utilization of the *Dicephalospora* spp. was rarely published maybe due to the minimal biomass in nature, difficulty of getting pure culture, and slow-growth if cultured.

During the examinations of helotialean fungi from China, three species fit well with the emended generic concept of *Dicephalospora* (Zhuang et al. 2016). However, new collections are found to differ from hitherto known species of *Dicephalospora*. To confirm their affinities and investigate their relationships with other species, phylogenetic analyses were conducted based on the internal transcribed spacers and 5.8S of nuclear ribosomal DNA (ITS).The results support their placement within the genus and their distinctions from any known species.

## Materials and methods

Specimens were collected, recorded, and photographed by a Canon PowerShot G16 digital camera in the field. Descriptions of gross morphology and substrate were according to field notes and photos. Dried apothecia were rehydrated with distilled water and sectioned at a thickness of 15−20 μm with a Yidi YD-1508A freezing microtome (Jinhua, China). Measurements were taken from longitudinal sections and squash mounts in lacto-phenol cotton blue solution using an Olympus BH-2 microscope (Tokyo, Japan). Iodine reactions of ascal apparatus were tested with or without 3% KOH solution pretreatment in Melzer’s reagent and Lugol’s solution (Baral 2009). Microscopic images were taken using a Canon G5 digital camera (Tokyo, Japan) attached to a Zeiss Axioskop 2 Plus microscope (Göttingen, Germany). Voucher specimens were deposited in the Herbarium Mycologicum Academiae Sinicae (**HMAS**). Names of the new species were formally registered in the database Fungal Names (http://www.fungalinfo.net/fungalname/fungalname.html).

Pure cultures were obtained from some specimens following the method provided by Webster and Weber (2001) and preserved in the State Key Laboratory of Mycology, Institute of Microbiology, Chinese Academy of Sciences.

Genomic DNA was extracted from dried apothecia or pure culture, using Plant Genomic DNA Kit (TIANGEN Biotech. Co., Beijing, China). ITS region was amplified and sequenced using the primer pair ITS1/ITS4 (White et al. 1990). PCR reactions had a final volume of 30 μl, containing 15 μl 2×Taq MasterMix (Beijing CWBiotech, China), 1.5 μl of each primer (10 mM), 2 μl DNA, and 10 μl deionized water. PCR reactions were carried out in an Applied Biosystems 2720 thermocycler (Foster City, CA, USA) under the following conditions: 94 °C for 5 min, followed by 35 cycles of 94 °C for 30 s, 53 °C for 30 s and 30 s at 72 °C, and a final extension of 72 °C for 10 min. The PCR products were purified and sequenced at Beijing Tianyi Huiyuan Bioscience and Technology, China.

Newly generated sequences were assembled and edited using BioEdit 7.0.5.3 (Hall 1999) or SeqMan (DNASTAR, Lasergene 7.1.0). The new sequences were deposited in GenBank and additional sequences were downloaded from GenBank (Table 1). *Lachnumpygmaeum* (Fr.) Bres. and *L.spartinae* S.A. Cantrel were chosen as outgroup taxa. The ITS sequence matrix was aligned and manually edited using BioEdit 7.0.5.3 (Hall 1999). Phylogenetic analyses were performed using maximum parsimony (MP) and neighbor-joining (NJ) methods with PAUP* 4.0b10 and parameters were set according to Zheng and Zhuang (2015). The topological confidence of the NJ and MP trees was assessed with bootstrap analysis using 1,000 replications, each with 10 replicates of random stepwise addition of taxa. The resulting trees were viewed via TreeView 1.6.6 (Page 1996).

**Table 1.** Sequences used in this study.

Species	Specimen/strain	ITS
*Chlorospleniumchlora* (Schwein.) M.A. Curtis	HMAS 266518	**MK425599**
	HMAS 279692	**MK425600**
*Ciboriabatschiana* (Zopf) N.F. Buchw.	CBS 312.37	KF859931
*Ciboriniafoliicola* (E.K. Cash & R.W. Davidson) Whetzel	1932.H	Z80892
*Dicephalosporaalbolutea* H.D. Zheng & W.Y. Zhuang	HMAS 279693	**MK425601**
*Dicephalosporaaurantiaca* (W.Y. Zhuang) W.Y. Zhuang & Z.Q. Zeng	HMAS 61850	DQ986486
*Dicephalosporachrysotricha* (Berk.) Verkley	ICMP:19950	KF727410
	ICMP:19952	KF727411
*Dicephalosporadentata* Xiao X. Liu & W.Y. Zhuang	HMAS 266694	KP204263
*Dicephalosporahuangshanica* (W.Y. Zhuang) W.Y. Zhuang & Z.Q. Zeng	HMAS 74836	DQ986485
	HMAS 81364	DQ986484
	HMAS 279694	**MK425602**
*Dicephalosporarufocornea* (Berk. & Broome) Spooner	HMAS 75518	DQ986480
	10106	KU668565
	HMAS 279695	**MK425603**
	HMAS 279696	**MK425604**
	HMAS 279697	**MK425605**
*Dicephalosporashennongjiana* H.D. Zheng & W.Y. Zhuang	HMAS 279698	**MK425606**
*Dicephalosporayunnanica* H.D. Zheng & W.Y. Zhuang	HMAS 279699	**MK425607**
	HMAS 279700	**MK425608**
	HMAS 279701	**MK425609**
*Hymenoscyphusfructigenus* (Bull.) Gray	CBS650.92	GU586933
	HMAS 75893	JX977144
*Lachnumpygmaeum* (Fr.) Bres.	ARON 2924.S	AJ430215
*Lachnumspartinae* S.A. Cantrel	SAP 138	AF422970
*Lambertellacorni-maris* Höhn.	CLX 3892	KC958560
	CLX 4075	KC958562
*Lanziaallantospora* (Dennis) Spooner	PRJ D804	AY755334
*Lanzialuteovirescens* (Roberge ex Desm.) Dumont & Korf	1823	KC533545
*Moellerodiscuslentus* (Berk. & Broome) Dumont	7818	KU668564
	10544	KU668566
*Moniliniafructicola* (G. Winter) Honey	MO-3D	JN001480
	RS10	JF325841
*Rutstroemiafirma* (Pers.) P. Karst.	2089.1	Z80893
	2089	KC533547
*Sclerotiniasclerotiorum* (Lib.) de Bary	2	KF148605
	6	KF148609

* Numbers in bold indicate sequences produced by this study.

## Results

### Phylogenetic analyses

**Figure 1.**
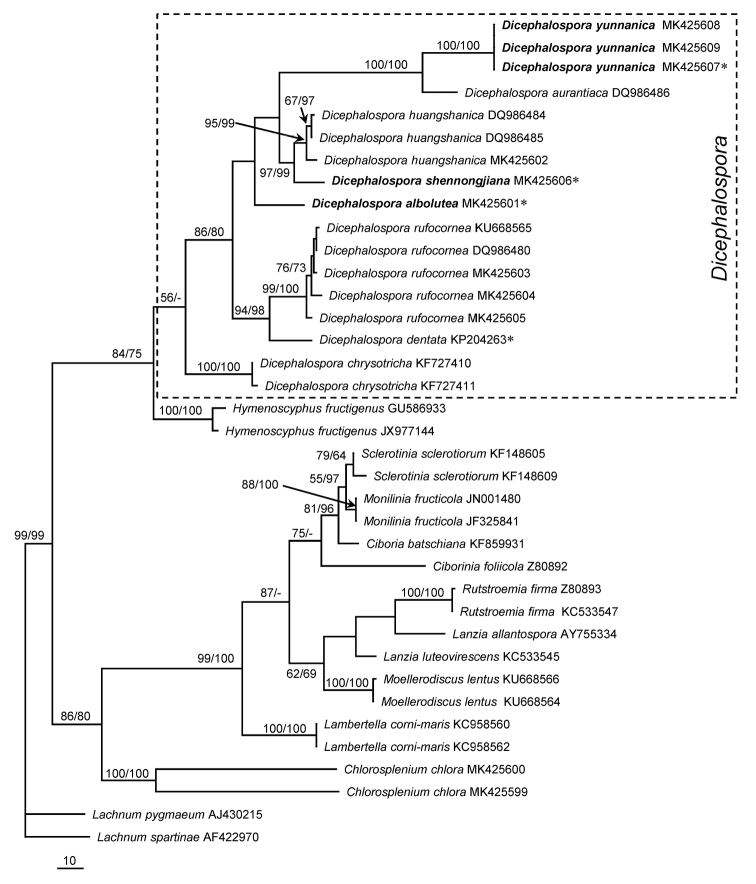
One of the MP trees inferred from ITS sequences. Bootstrap support values (≥50%) of MP and NJ are shown at nodes from left to right. New proposed species are shown in bold. New species are in bold. Sequences derived from holotypes are marked with an asterisk (*).

The ITS dataset included 37 sequences from eight *Dicephalospora* species, 11 related fungi and two outgroup taxa. The final alignment resulted in 634 characters including gaps, of which 252 were parsimony-informative, 38 were variable and parsimony-uninformative, and 344 were constant. In the MP analysis, eight most parsimonious trees were generated (tree length = 790, consistency index = 0.5899, homoplasy index = 0.4101, retention index = 0.8126, rescaled consistency index = 0.4793) and one of them was shown in Figure 1. MP and NJ bootstrap proportions (BP) greater than 50% were labeled at the nodes.

From topology of the phylogenetic tree (Fig. 1), *Dicephalospora* species clustered together with a medium supporting value (56% MPBP). The three putative new species were clearly distinct from the known and sequenced species of the genus. *Dicephalosporaalbolutea* appeared as an independent lineage distinct from any other members of the genus. *Dicephalosporashennongjiana* was resolved as a sibling species of *D.huangshanica* (97% MPBP and 99% NJBP). ITS sequences of the three collections of *D.yunnanica* were identical and formed a well-supported group with *D.aurantiaca* (100% MPBP and 100% NJBP).

### Taxonomy

#### Dicephalospora albolutea

Taxon classificationFungiHelotialesSclerotiniaceae

H.D. Zheng & W.Y. Zhuang sp. nov.

Figure 2

##### Etymology.

The specific epithet refers to the color of apothecia.

##### Holotype.

CHINA. Yunnan Province, Binchuan County, Jizu Mountain, alt. 2500 m, on rotten leaf veins, 21 September 2017, H.D. Zheng, X.C. Wang, Y.B. Zhang & Y. Zhang 11613 (HMAS 279693, ITS GenBank accession number: MK425601).

##### Description.

*Apothecia* scattered, discoid, stipitate, with even margin, 1−2.5 mm in diameter; hymenium surface cream to yellowish white; receptacle surface concolorous. *Ectal excipulum* of textura prismatica, 20−70 μm thick, cells somewhat thick- and glassy-walled, 16.5−40 × 5.5−11 μm. *Medullary excipulum* of textura porrecta and textura intricata, 25−275 μm thick, hyphae hyaline, thin-walled, 2.5−5 μm wide. *Subhymenium* not distinguishable. *Hymenium* 165−175 μm thick. *Asci* unitunicate, arising from simple septa, 8-spored, cylindric-clavate, J+ in Melzer’s reagent and Lugol’s solution without KOH pretreatment, visible as two blue lines, 140−156 × 9.5−10.5 μm. *Ascospores* sausage-shaped to subfusoid, with anterior end rounded and posterior end narrower, slightly curved, aseptate, hyaline, smooth, lacking a gel cap at each end, multiguttulate, with a dark-stained area when mounted in cotton blue solution, biseriate, 26−31 × 3.8−5.0 μm. *Paraphyses* filiform, straight, slightly enlarged at apex, hyaline, septate, 3−3.5 μm broad at upper portion and 1.5−2 μm below, equal to or very slightly exceeding the asci.

**Figure 2.**
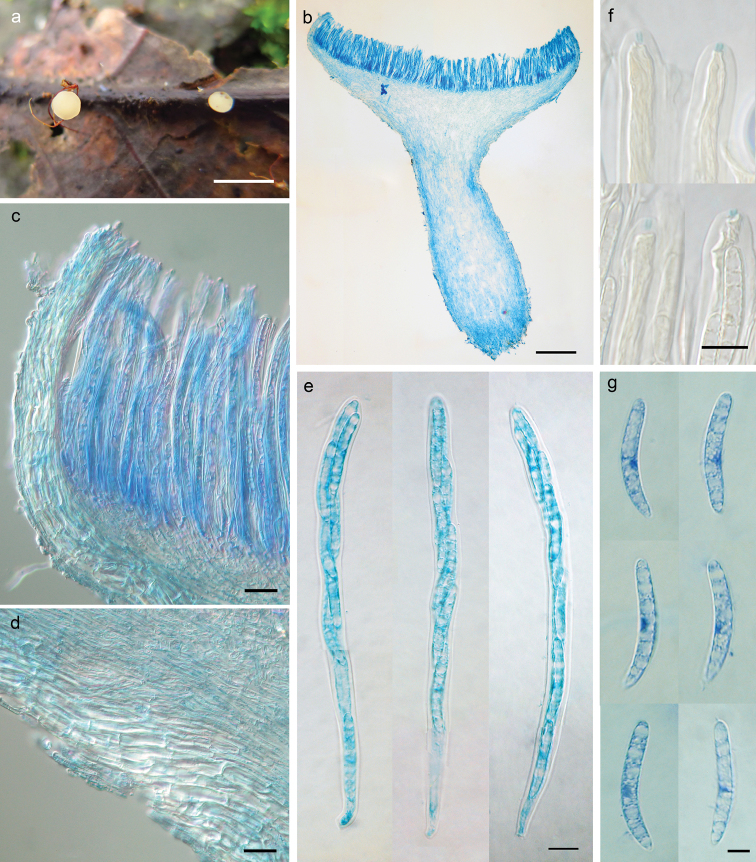
*Dicephalosporaalbolutea* (HMAS 279693, **holotype**). **a** fresh apothecia on natural substrate **b** longitudinal section of apothecium **c** structure of margin and hymenium **d** structure of flank **e** asci **f** IKI reaction of apical rings **g** ascospores. Mouting media: **b–e, g** lacto-phenol cotton **f** lugol’s solution. Scale bars: 5 mm (**a**); 200 μm (**b**); 20 μm (**c, d**); 10 μm (**e, f**); 5 μm (**g**).

##### Notes.

The diagnostic features of *D.albolutea* are cream to yellowish white apothecia and sausage-shaped ascospores. The apothecial color of earlier known *Dicephalospora* species varied from yellow, orange, red to dark, but never as pale as that in *D.albolutea*. *Dicephalosporacalochroa* (Syd. & P. Syd.) Spooner is somewhat similar in length of asci and ascospores, but differs by vivid orange apothecia, wider asci (125−150 × 12−15 μm) and ascospores (20−25 × 6−8 μm), which are pointed at both ends (Spooner 1987). *Dicephalosporaalbolutea* differs from any investigated species by at least 45 bp in sequences of ITS region, and appeared as an independent lineage in the phylogenetic tree (Fig. 1), which further confirmed its distinction from others in the group.

#### Dicephalospora shennongjiana

Taxon classificationFungiHelotialesSclerotiniaceae

H.D. Zheng & W.Y. Zhuang sp. nov.

Figure 3

##### Etymology.

The specific epithet refers to the type locality of the fungus.

##### Holotype.

CHINA. Hubei Province, Shennongjia, Shennongyuan, alt. 2250 m, on stromatized dead vine, 15 Sept 2014, H.D. Zheng, Z.Q. Zeng, W.T. Qin & K. Chen 9589 (HMAS 279698, ITS GenBank accession number: MK425606).

##### Description.

*Apothecia* scattered, discoid to flat, stipitate, with even margin, 0.5−0.8 mm in diameter; hymenium surface greenish yellow; receptacle surface slightly darker. *Ectal excipulum* of textura prismatica, 15−40 μm thick, cells hyaline to pale brownish, somewhat thick- and glassy-walled, 10−20 × 4−11 μm. *Medullary excipulum* of textura intricata, 25−110 μm thick, hyphae hyaline, thin-walled, 2−4 μm wide. *Subhymenium* about 15 μm thick. *Hymenium* 170−180 μm thick. *Asci* arising from simple septa, unitunicate, 8-spored, clavate, J+ in Melzer’s reagent and Lugol’s solution without KOH pretreatment, visible as two blue lines, 130−150 × 14−16.5 μm. *Ascospores* elliptical-subfusoid, aseptate, hyaline, smooth, lacking a gel cap at each end, multiguttulate, with a dark-stained area when mounted in cotton blue solution, uniseriate, 19−22 × 7−8.8 μm. *Paraphyses* filiform, slightly enlarged at apex, hyaline, septate, branched and tangled near apex, 3−3.5 μm broad at upper portion and 1.5−2 μm below, exceeding the asci by 10−20 μm.

**Figure 3.**
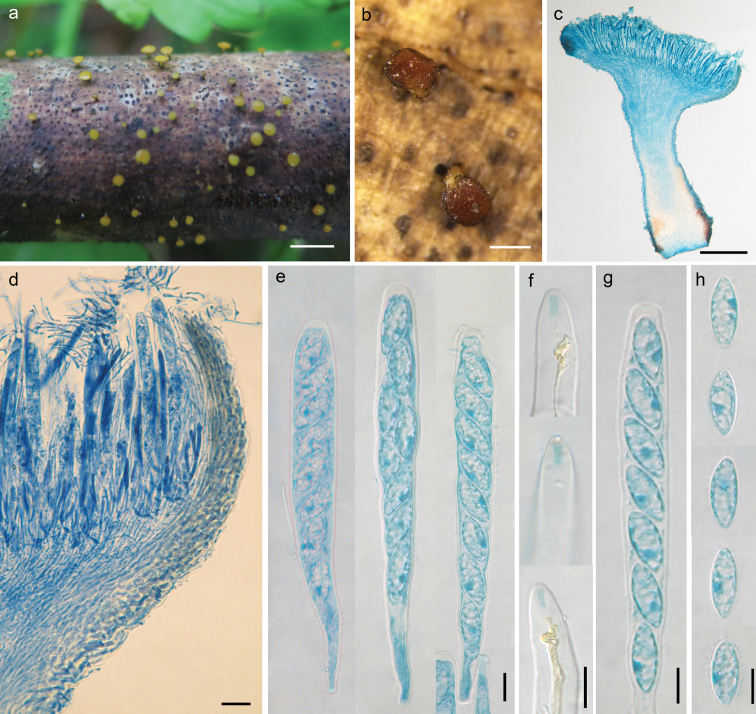
*Dicephalosporashennongjiana* (HMAS 279698, **holotype**) **a** fresh apothecia on natural substrate **b** dried apothecia **c** longitudinal section of apothecium **d** structure of margin, flank and hymenium **e** asci **f** IKI reaction of apical rings **g** ascospores in an ascus **h** ascospores. Mouting media: **c–e, g, h** lacto-phenol cotton **f** fugol’s solution. Scale bars: 2 mm (**a**); 0.4 mm (**b**); 200 μm (**c**); 20 μm (**d**); 10 μm (**e–h**).

##### Notes.

The new species can be distinguished from other species by shape of ascospores and tangled paraphyses apices. *Dicephalosporadamingshanica* has a similarly shaped ascospore, but larger (22−32 × 9−12.7 μm), and with a hyaline mucilaginous cap at both ends (Zhuang 1999). Phylogenetically, *D.shennongjiana* is closely related to *D.huangshanica*, but the latter differs by red apothecia, smaller asci (89−96 × 9.5−11 μm), fusoid ascospores (18−26 × 4−5 μm) (Zhuang 1995a), and 22 bp divergence in ITS region.

#### Dicephalospora yunnanica

Taxon classificationFungiHelotialesSclerotiniaceae

H.D. Zheng & W.Y. Zhuang sp. nov.

Figure 4

##### Etymology.

The specific epithet refers to the type locality of the fungus.

##### Holotype.

CHINA. Yunnan Province, Maguan County, Dabao Village, alt. 1565 m, on rotten leaf rachis, 13 August 2016, X.H. Wang, S.H. Li, H.D. Zheng & S.C. Li YN16-108 (HMAS279699, ITS GenBank accession number: MK425607).

##### Description.

*Apothecia* scattered, discoid, stipitate, with even margin, 0.8−2.0 mm in diameter; hymenium surface bright yellow to orange; receptacle surface paler. *Ectal excipulum* of textura prismatica, 22−60 μm thick, cells hyaline, somewhat thick- and glassy-walled, 7−20 × 5−7 μm. *Medullary excipulum* of textura intricata, 30−230 μm thick, hyphae thin-walled, 2−5 μm wide. *Subhymenium* not distinguishable. *Hymenium* 100−115 μm thick. *Asci* arising from simple septa, unitunicate, 8-spored, cylindric-clavate, J+ in Melzer’s reagent and Lugol’s solution without KOH pretreatment, visible as two faint blue lines, 85−100 × 7.5−8.5 μm. *Ascospores* fusoid, aseptate, with one side very slightly flattened and pointed at ends, hyaline, smooth, lacking a gel cap at each end, multiguttulate, biseriate, 16.5−25.3 × 3.3−3.5 μm. *Paraphyses* filiform, slightly enlarged at apex, straight or sometimes slightly curved at the apical portion, hyaline, septate, 2.5−4 μm broad at upper portion and 1.5−2 μm below, slightly exceeding the asci by about 5 μm.

**Figure 4.**
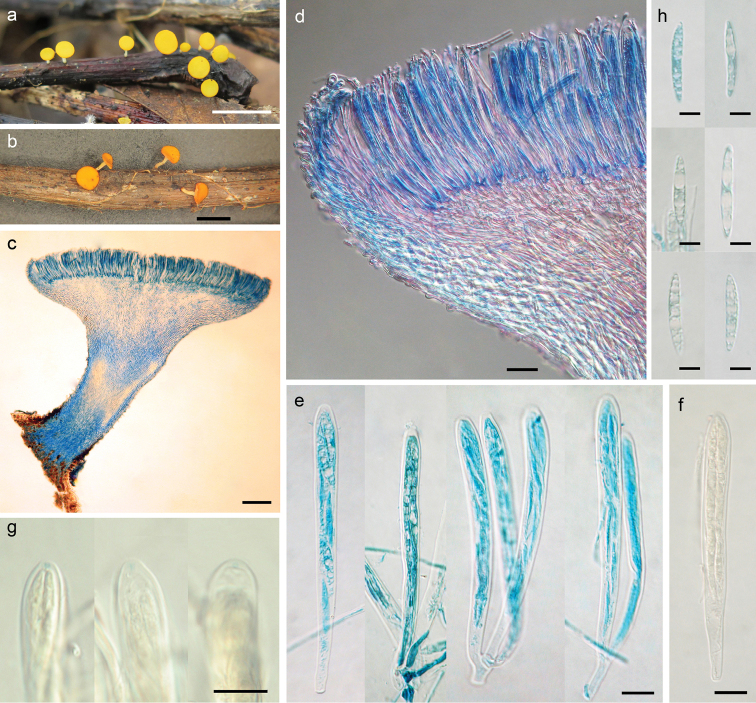
*Dicephalosporayunnanica* (HMAS 279699, **holotype**) **a** fresh apothecia on natural substrate **b** dried apothecia **c** longitudinal section of apothecium **d** structure of margin, flank and hymenium **e, f** asci **g** IKI reaction of apical rings **h** ascospores. Mouting media: **c–e, h** lacto-phenol cotton **f, g** lugol’s solution. Scale bars: 5 mm (**a**); 2 mm (**b**); 200 μm (**c**); 20 μm (**d**); 10 μm (**e–g**); 5 μm (**h**).

##### Additional specimens examined.

CHINA. Yunnan Province, Maguan County, Xiaobaozi Town, alt. 1550 m, on rotten leaf rachis, 13 August 2016, X.H. Wang, S.H. Li, H.D. Zheng & S.C. Li YN16-135 (HMAS 279700); Maguan County, Pojiao Village, alt. 1450 m, on rotten leaf rachis, 14 August 2016, X.H. Wang, S.H. Li, H.D. Zheng & S.C. Li YN16-165 (HMAS 279700).

##### Notes.

*Dicephalosporayunnanica* shares similar gross morphology with and appeared to be sister of *D.aurantiaca* in the phylogenetic tree (Fig. 1). However, *D.aurantiaca* has larger asci (93−103 × 9.7−10.5 μm) and ascospores (21−26 × 4−4.8 μm), as well as obviously curved paraphysis apices (Zhuang 1995a; Zhuang et al. 2016). Concerning the DNA sequence data, the three collections of *D.yunnanica* share exactly the same sequences, while the closest species *D.aurantiaca* showed 75 bp divergence (including 32 gaps) for ITS.

### A taxonomic key to the known species of *Dicephalospora*

**Table:** 

1	Receptacle surface covered with hairs	*** D. chrysotricha ***
–	Receptacle surface without hairs	**2**
2	Apothecial margin dentate	*** D. dentata ***
–	Apothecial margin even	**3**
3	Hymenium surface cream to yellowish white when fresh	*** D. albolutea ***
–	Hymenium surface darker in color	**4**
4	Hymenium surface red or dark red	*** D. huangshanica ***
–	Hymenium surface lacking of a red tint	**5**
5	Paraphyses with darkly pigmented contents	*** D. phaeoparaphysis ***
–	Paraphyses without darkly pigmented contents	**6**
6	Ascospores with a gel cap at each end	**7**
–	Ascospores lacking of a gel cap at each end	**10**
7	Ascospores 9−12.7 μm wide	*** D. damingshanica ***
–	Ascospores less than 9 μm wide	**8**
8	Asci J−, ascospores 20−28 × 4.5−5.7 μm	*** D. pinglongshanica ***
–	Asci J+	**9**
9	Ascospores 23−27(−29) × 6.5−7.5 μm	*** D. calochroa ***
–	Ascospores (27−)32−39 × 4−5.5(−6) μm	*** D. rufocornea ***
10	Ascospores constricted in the middle, 20−27 × 4−5 μm	*** D. contracta ***
–	Ascospores not constricted in the middle	**11**
11	Ascospores 19−22 × 7−8.8 μm	*** D. shennongjiana ***
–	Ascospores less than 7 μm wide	**12**
12	Ascospores 16.5−25.3 × 3.3−3.5 μm, paraphyses straight	*** D. yunnanica ***
–	Ascospores 21−26 × 4−4.8 μm, paraphyses curved at apex	*** D. aurantiaca ***


## Discussion

Identification of *Dicephalospora* species is mainly based on morphological features, such as color of apothecia, anatomic structure, and characteristics of asci and ascospores. DNA sequence data are sometimes considered, which play an important role in the delineation of fungal species (Hibbett et al. 2016; Jeewon and Hyde 2016). In the present study, three new species were introduced based on morphology and ITS phylogeny. So far, the genus comprises 13 species, of which 12 have been reported from China. *Dicephalosporachrysotricha* (Berk.) Verkley originally described from, and endemic to, New Zealand, is the only exception and known only from the type locality (Verkley 2004).

In the phylogenetic analyses, only some species possessing fusoid to sausage-shaped and elliptic-subfusoid ascospores were involved due to limitation of the available sequences. The ITS barcodes seem to be useful for distinguishing *Dicephalospora* species, as they grouped as well-separated clades (Fig. 1). Seven of the eight species were together receiving moderate statistic supports (86% MPBP and 80% BIPP) and formed the core group. However, *D.chrysotricha* joined them as a distantly separated lineage with very low support (Fig. 1, 56% MPBP). *Dicephalosporachrysotricha* is distinct from any other taxa of the genus in having hair-like projections on receptacle surface. *Dicephalosporachrysotricha* was previously treated as a member of *Trichopeziza* Fuckel (Saccardo 1889) and then *Chlorosplenium* Fr. (Dennis 1961). The transfer of this species to *Dicephalospora* might have been because of presence of polar mucilaginous caps of ascospores and the more or less similar ectal excipulum structure except for hairs (Verkley 2004). However, it does not fit well the generic concept of *Dicephalospora*. Further study is required to clarify the taxonomic position of this fungus.

As to the phylogenetic position of *Dicephalospora*, Figure 1 shows its close relationship with *Hymenoscyphus* Gray, which agrees with the treatment of Wijayawardene et al. (2017). Similar results were also achieved in other recent studies (Han et al. 2014; Zhao et al. 2016). In the phylogenetic study of Hyaloscyphaceae and related helotialean cup-fungi, *D.huangshanica* and *D.rufocornea* grouped together with some genera of Helotiaceae, such as *Hymenoscyphus*, *Crocicreas* Fr. and *Cudoniella* Sacc., as a highly supported clade in the maximum-likelihood tree inferred from combined sequence data of ITS, the large subunit nrDNA gene (LSU), the second largest subunit of RNA polymerase II gene (RPB2), and mitochondrial small subunit (mtSSU) (Han et al. 2014). Zhao et al. (2016) carried out phylogenetic analyses of *Lambertella* Höhn. and allied genera including *Dicephalospora* and *Hymenoscyphus*, as inferred from ITS, LSU and RPB2 sequence data. In their phylogenetic trees, *D.rufocornea* was also associated with the clade consisting of *Hymenoscyphus* species. In view of the above results, close relationship of *Dicephalospora* with genera of Helotiaceae is obvious. Comprehensive work containing more genera and more genes are required to obtain an accurate conclusion on phylogenetic placement of *Dicephalospora*.

## Supplementary Material

XML Treatment for Dicephalospora albolutea

XML Treatment for Dicephalospora shennongjiana

XML Treatment for Dicephalospora yunnanica
